# Can coronavirus disease 2019 affect male fertility or cause spontaneous abortion? A two-sample Mendelian randomization analysis

**DOI:** 10.1515/biol-2025-1188

**Published:** 2025-10-30

**Authors:** Yufeng Liang, Xueshan Ma, Chunli Liu, Xiuqing He, Tao Liu, Xiaoming Niu

**Affiliations:** Department of Reproduction and Genetics, The Affiliated Taian City Central Hospital of Qingdao University, No. 29 Long Tan Road, Tai’an, Shandong, 271000, P. R. China; Department of Gynecology, The Affiliated Taian City Central Hospital of Qingdao University, Tai’an, Shandong, 271000, P. R. China

**Keywords:** COVID-19, Mendelian randomization, spontaneous abortion, male infertility, causal relationship

## Abstract

To investigate the causal relationship between two adverse reproductive outcomes (male infertility and spontaneous abortion) and COVID-19 infection using Mendelian randomization (MR) analysis. A two-sample MR study was conducted to examine potential causal links between COVID-19 infection severity and reproductive outcomes, using large-scale genome-wide association study (GWAS) summary statistics from European-ancestry populations. GWAS summary statistics were analyzed for COVID-19 phenotypes (infection: *n* = 1,683,769; hospitalized: *n* = 1,557,411; very severe respiratory-confirmed: *n* = 1,388,342; critical illness: *n* = 10,056) and reproductive outcomes (spontaneous abortion: *n* = 98,453; male infertility: *n* = 73,479). Causal estimates were calculated using inverse variance weighted (IVW), weighted median, MR-Egger regression, and weighted mode methods. IVW analysis revealed no significant association between genetic susceptibility to COVID-19 infection and male infertility (odds ratio [OR] = 0.7668; 95% confidence interval [CI]: 0.3798–1.5484; *p* = 0.4590) or spontaneous abortion (OR = 0.9936; 95% CI: 0.8066–1.2241; *p* = 0.8518). Similar null associations between COVID-19 severity phenotypes (hospitalized, very severe respiratory-confirmed, and critical illness) and male infertility or spontaneous abortion were observed. Sensitivity analyses using alternative methods confirmed the absence of pleiotropy and heterogeneity. This two-sample MR analysis provides robust evidence against a causal relationship between COVID-19 and increased risks of male infertility or spontaneous abortion.

## Graphical abstract

Mendelian randomization (MR) analysis reveals no causal effect of COVID-19 infection on male infertility or spontaneous abortion.

**Figure:**
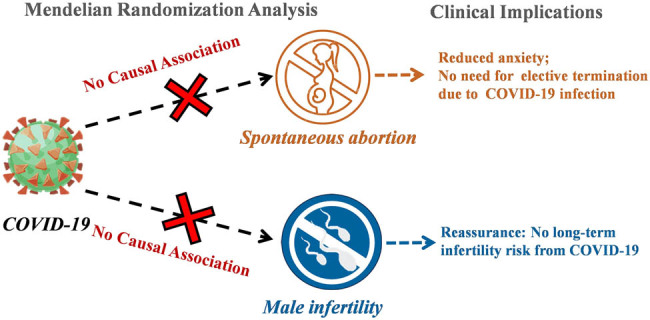

## 1 Introduction

The severe acute respiratory syndrome coronavirus-2 (SARS-CoV-2) was first identified in December 2019 during an outbreak of fatal pneumonia in Wuhan, China [1]. Subsequently, the World Health Organization officially named the disease caused by this novel coronavirus “coronavirus disease-2019 (COVID-19).” The ongoing COVID-19 pandemic has affected millions globally and continues to represent a significant public health challenge [2]. After recovery from the acute infection, up to 30% of COVID-19 survivors may develop post-COVID-19 syndrome [3], which is characterized by persistent symptoms and long-term sequelae.

The global impact of COVID-19 goes beyond respiratory complications, potentially affecting multiple organ systems. Although pulmonary involvement remains the predominant manifestation, an increasing body of evidence indicates that COVID-19 may trigger various systemic abnormalities, including potential testicular dysfunction [4]. Given the multi-organ impact of COVID-19, concerns have been raised about its potential effects on reproductive health. Recent studies have detected SARS-CoV-2 in semen samples, raising concerns about possible paternal transmission [5]. Several comprehensive reviews have explored the potential testicular damage associated with COVID-19 infection and its possible implications for male fertility [6]. A 2024 systematic review of 135 studies highlighted that SARS-CoV-2 infection transiently reduces sperm motility and morphology, though these parameters often recover within spermatogenic cycles, suggesting acute inflammatory effects rather than permanent damage [7]. Conversely, another 2024 systematic analysis reported hormonal alterations and impaired semen quality in convalescent men, yet found no viral RNA in semen, underscoring unresolved pathophysiological mechanisms [8]. Some researchers have proposed that mature spermatozoa may interact with the virus and potentially serve as viral vectors [9]. The primary cause of male infertility is compromised sperm quality, and COVID-19 may additionally disrupt the hypothalamic–pituitary–gonadal axis, which could further contribute to fertility impairment [6]. These globally heterogeneous findings highlight the urgency of clarifying causal relationships to inform public health policies and alleviate undue reproductive anxiety worldwide.

Similarly, as the global prevalence of COVID-19 continues to rise, data on its impact on pregnancy outcomes remain limited. Early meta-analyses suggested elevated risks of adverse pregnancy outcomes, including preterm birth, preeclampsia, cesarean delivery, and neonatal mortality [10]. However, large-scale cohort studies reported conflicting evidence on spontaneous abortion [11,12]. A systematic review of 42 studies (*n* = 438,548) found no significant increase in spontaneous abortion risk [11], whereas a retrospective cohort study (*n* = 193) documented nine cases of spontaneous abortion without establishing a causal link [12]. The paucity of robust data regarding COVID-19-associated spontaneous abortions during the first trimester hinders our understanding of early pregnancy complications. Notably, a case-control study (*n* = 225) confirmed COVID-19 does not predict early pregnancy loss [13], yet this inconsistency persists. This information gap has fueled public anxiety and the potential spread of media misinformation, which may influence pregnant women to consider elective termination without valid medical reasons [14]. Therefore, it is of paramount importance to determine the potential role of COVID-19 infection in spontaneous abortion.

Observational studies investigating the association between COVID-19 and reproductive outcomes are inherently constrained by potential confounding factors and reverse causality. Randomized controlled trials are unethical in this context. Mendelian randomization (MR) overcomes these limitations by using genetic variants as instrumental variables [15,16]. This approach leverages the random assortment of alleles to mimic a randomized trial, providing more robust causal inferences [15,16]. In this study, we employed a two-sample MR design to investigate the potential causal effects of COVID-19 on male infertility and spontaneous abortion. By utilizing genetic variants associated with COVID-19 as instrumental variables, we aimed to provide robust evidence regarding the potential impact of COVID-19 on reproductive outcomes. We hypothesized that there was no significant causal association between COVID-19 infection and an increased risk of male infertility or spontaneous abortion. Key findings and clinical implications were shown in graphical abstract.

## 2 Methods

### 2.1 Data sources

This study utilized publicly available summary-level data from large-scale genome-wide association studies (GWAS) consortia. The authors did not carry out any direct patient recruitment or selection procedures. Genetic association summary statistics for four COVID-19 severity phenotypes were retrieved from the Integrative Epidemiology Unit Open GWAS project (https://gwas.mrcieu.ac.uk/). For this MR study, the exposure datasets were defined by COVID-19 (ebi-a-GCST011073), COVID-19 hospitalized (ebi-a-GCST011083), COVID-19 very severe respiratory confirmed (ebi-a-GCST011075), and COVID-19 critical illness (ebi-a-GCST90013414). The outcome GWAS summary statistics for male infertility (finn-b-N14_MALEINFERT) and spontaneous abortion (finn-b-O15_ABORT_SPONTAN) were obtained from the FinnGen consortium Release R10 (https://www.finngen.fi/en). All exposure and outcome cases were of European descent. Detailed characteristics of the exposures and outcomes of GAWS are shown in Table 1.

**Table 1:** Characteristics of the GWASs used for the MR analyses

GWAS ID	Phenotype	Sample size	Cases (*n*)	Controls (*n*)	SNPs (*n*)	Population
ebi-a-GCST011073	COVID-19	1,683,748	38,984	1,644,784	8,660,177	European
ebi-a-GCST011083	COVID-19 (hospitalized)	1,557,411	8,316	1,549,095	8,110,403	European
ebi-a-GCST011075	COVID-19 (very severe respiratory-confirmed)	1,388,342	5,101	1,383,241	9,739,225	European
ebi-a-GCST90013414	COVID-19 (critical illness)	10,056	1,676	8,380	4,264,568	European
finn-b-O15_ABORT_SPONTAN	Spontaneous abortion	98,453	9,113	89,340	16,379,138	European
finn-b-N14_MALEINFERT	Male infertility	73,479	680	72,799	16,377,329	European

GWAS, genome-wide association study; SNP, single-nucleotide polymorphism.

### 2.2 Genetic instrument selection for COVID-19 phenotypes

**Figure 1:**
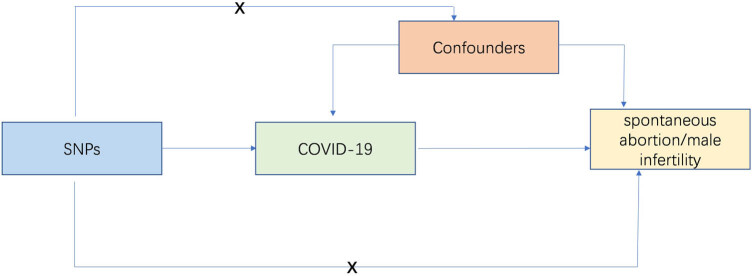
The core instrumental variable assumptions of MR study. SNP, single-nucleotide polymorphisms.

An overview of the study design was presented in Figure 1. COVID-19 showed a strong association with seven single-nucleotide polymorphisms (SNPs; *p* < 5 × 10^−8^, linkage disequilibrium *r* ^2^ < 0.01). Both COVID-19 hospitalization and COVID-19 with very severe respiratory confirmation were associated with eight SNPs, whereas COVID-19 critical illness was related to five SNPs (*p* < 5 × 10^−8^, linkage disequilibrium *r* ^2^ < 0.01). Detailed information about the SNPs was provided in Table 2. All SNPs exhibited *F*-statistics >10, indicating strong instrument validity.

**Table 2:** Details of the genetic instrumental variables (SNPs) associated with each COVID-19 severity phenotype

SNP	Effect allele	Other allele	*β*	MAF	SE	*p*-value	*R* ^2^	*F*
**COVID-19**								
rs10936744	T	C	−0.0626	0.3588	0.0100	3.51 × 10^−10^	0.0018	3034.522
rs12482060	G	C	0.06195	0.3375	0.0105	3.96 × 10^−09^	0.0017	2884.842
rs17078348	G	A	0.09208	0.0997	0.0161	1.20 × 10^−08^	0.0015	2559.18
rs2271616	T	G	0.15634	0.1181	0.0150	3.61 × 10^−25^	0.0051	8529.123
rs4971066	G	T	−0.0767	0.1777	0.0134	1.02 × 10^−08^	0.0017	2894.5
rs643434	A	G	0.1013	0.371	0.0101	1.29 × 10^−23^	0.0049	8025.465
rs757405	A	T	0.06892	0.7092	0.0107	1.64 × 10^−10^	0.0020	3292.98
**COVID-19 (hospitalized)**								
rs10860891	A	C	−0.1828	0.8547	0.0331	3.43 × 10^−08^	0.0083	13034.19
rs111837807	C	T	0.2155	0.1004	0.0343	3.22 × 10^−10^	0.0084	13175.56
rs13050728	C	T	−0.1861	0.6382	0.0239	6.72 × 10^−15^	0.0160	25313.54
rs1859330	A	G	0.1561	0.6979	0.0226	4.91 × 10^−12^	0.0103	16168.43
rs2109069	A	G	0.1873	0.322	0.0236	1.96 × 10^−15^	0.0153	24226.89
rs35081325	T	A	0.5462	0.0859	0.0354	7.93 × 10^−54^	0.0468	76527.59
rs41264915	G	A	−0.2049	0.0817	0.0358	1.02 × 10^−08^	0.0063	9870.101
**COVID-19 (very severe respiratory-confirmed)**								
rs10860891	A	C	−0.2395	0.8855	0.0397	1.64 × 10^−09^	0.0116	16338.49
rs111837807	C	T	0.2945	0.0996	0.0428	5.66 × 10^−12^	0.0156	21938.16
rs13050728	C	T	−0.2001	0.6627	0.0286	2.44 × 10^−12^	0.0179	25304.5
rs2109069	A	G	0.2566	0.3287	0.0281	6.12 × 10^−20^	0.0291	41549.14
rs2237698	T	C	0.2366	0.0897	0.0397	2.41 × 10^−09^	0.0091	12809.17
rs2384074	T	C	0.1982	0.6756	0.0282	2.10 × 10^−12^	0.0172	24324.66
rs35081325	T	A	0.6262	0.0753	0.0445	5.75 × 10^−45^	0.0546	80192.84
rs77534576	T	C	0.45975	0.0347	0.1255	8.52 × 10^−10^	0.0142	19941.36
**COVID-19 (critical illness)**								
rs10735079	A	G	0.2578	0.3514	0.0457	1.65 × 10^−08^	0.0303	314.1043
rs143334143	A	G	0.6151	0.0823	0.0716	8.82 × 10^−18^	0.0572	609.4227
rs2109069	A	G	0.3056	0.2993	0.0440	3.98 × 10^−12^	0.0392	409.8913
rs2236757	G	A	−0.2511	0.2911	0.0461	5.00 × 10^−08^	0.0260	268.6214
rs73064425	T	C	0.7628	0.0695	0.0670	4.77 × 10^−30^	0.0753	818.2214

SNP, single-nucleotide polymorphisms; *β*, effect size; MAF, minor allele frequency; SE, standard error of *β*; *R* ^2^, the proportion of the variability of the exposure explained by instrumental variables.

### 2.3 Outcome data acquisition and harmonization

For each COVID-19-associated SNP, effect sizes and standard errors were extracted. Incompatible alleles and palindromic SNPs were excluded to ensure strand alignment.

### 2.4 Confounding SNP exclusion

To control for potential confounding factors, the PhenoScanner database (http://www.phenoscanner.medschl.cam.ac.uk) was employed to identify secondary phenotypes associated with COVID-19-related SNPs. SNPs were excluded if they were significantly associated with known confounders of reproductive outcomes, such as thrombophilia, autoimmune diseases, and endocrine disorders [17,18,19,20]. Specifically, rs643434, associated with thrombosis (*p* = 7 × 10^−63^) and deep vein thrombosis (*p* = 1 × 10^−173^), and rs111837807, linked to type 1 diabetes (*p* = 2 × 10⁻^11^), type 2 diabetes (*p* = 7 × 10⁻^20^), and hypothyroidism (*p* = 1 × 10⁻^10^), met these criteria and were excluded prior to conducting the MR analysis. The remaining SNPs showed no significant association with confounders of male infertility or spontaneous abortion.

### 2.5 Statistical analysis

The primary causal estimates for the relationship between COVID-19 and male infertility or spontaneous abortion were derived using the inverse variance weighted (IVW) method, which operates under the assumption of no horizontal pleiotropy. The main findings were based on an inverse variance-weighted meta-analysis of the Wald ratios for individual SNPs, predicated on the assumption that no unmeasured confounders or alternative pathways influenced the outcomes through the genetic instruments [21]. To complement the IVW estimates, MR-Egger regression, weighted median, and weighted mode methods were employed. These methods served as robustness checks to validate the primary findings.

**Figure 2:**
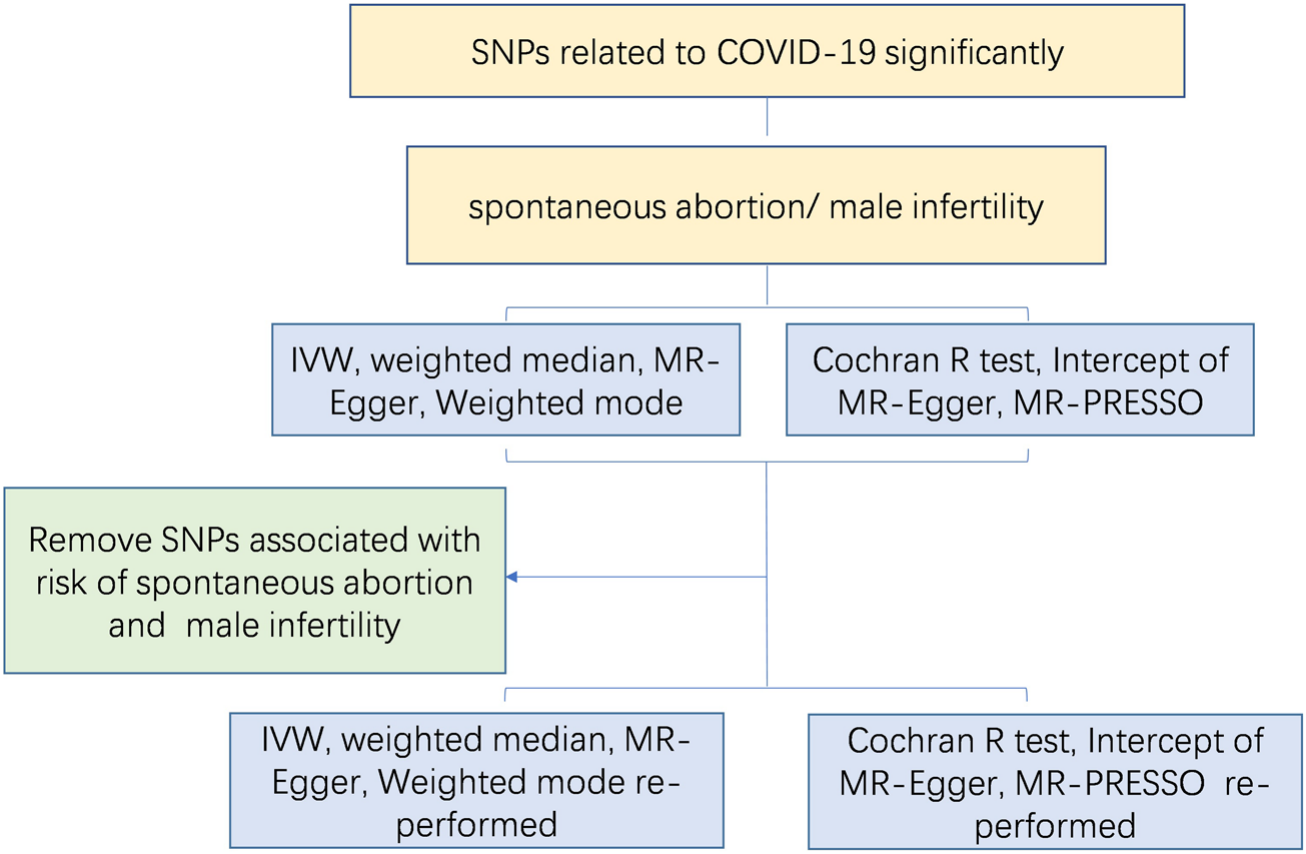
MR study methodology to reveal the causative influence of coronavirus disease-2019 (COVID-19) on spontaneous abortion and male infertility. IVW, inverse variance weighted; MR, Mendelian randomization; MR-PRESSO, MR Pleiotropy RESidual Sum and Outlier; SNP, single-nucleotide polymorphisms.


Sensitivity analyses were carried out to evaluate potential pleiotropy and heterogeneity, as their presence could significantly bias the MR estimates if not properly accounted for. Horizontal pleiotropy was assessed using Cochran’s *Q* statistic derived from the IVW method, which tests for heterogeneity among the genetic instruments. Directional pleiotropy was evaluated through the intercept term in MR-Egger regression, with a *p*-value of <0.05 indicating its presence [22]. Furthermore, the MR-Pleiotropy Residual Sum and Outlier (MR-PRESSO) method was applied to detect and rectify horizontal pleiotropy by identifying and eliminating outliers [23]. The MR-PRESSO procedure comprised three sequential steps: identification of horizontal pleiotropy, removal of outlier SNPs to correct for pleiotropic effects, and comparison of causal estimates before and after outlier removal to determine statistically significant differences. MR-Egger regression provides more precise and less biased estimates compared to IVW method when the proportion of genetic variants exhibiting horizontal pleiotropy was below 10% [24]. A leave-one-out analysis was performed to evaluate whether the MR estimates were disproportionately influenced by any single SNP. The MR study methodology was shown in Figure 2. All analyses were performed using MRPRESSO (version 0.6.4) and the TwoSampleMR (version 0.6.4) package in R (version 4.3.1).

**Ethical approval:** As this study involved the reanalysis of previously obtained and published data, no additional ethical approval was required.

## 3 Results

### 3.1 Impact of COVID-19 on spontaneous abortion

After excluding SNPs associated with thrombus formation (rs643434), hypothyroidism/diabetes (rs111837807), palindromic SNPs with intermediate allele frequencies, and incompatible alleles, the final counts of SNPs retained for each COVID-19 phenotype in the MR analyses were as follows: (1) for COVID-19 infection, six SNPs were retained; (2) for COVID-19 hospitalized, six SNPs were retained; (3) for COVID-19 very severe respiratory-confirmed, seven SNPs were retained; and (4) for COVID-19 critical illness, five SNPs were retained. We observed no significant association between COVID-19 infection and spontaneous abortion risk (IVW: odds ratio [OR] = 0.9936, 95% confidence interval [CI]: 0.8066–1.2241, *p* = 0.8518; Table 3). Consistent null associations were found across all COVID-19 severity phenotypes: hospitalized: OR = 1.0031, 95% CI = 0.9343–1.0770, *p* = 0.9314; very severe respiratory-confirmed: OR = 1.0012, 95% CI = 0.9478–1.0576, *p* = 0.9656; critical illness: OR = 1.0009, 95% CI = 0.9557–1.0481, *p* = 0.9703 (Table 3).

**Table 3:** MR estimates of the causal effect of COVID-19 phenotypes on the risk of spontaneous abortion

Outcome	Method	OR	95% CI	*p*-value
COVID-19	IVW	0.9936	0.8066–1.2241	0.8518
	MR Egger	1.6410	0.7899–3.4092	0.2087
	Weighted median	1.1349	0.8721–1.4767	0.5074
	Weighted mode	1.1799	0.8013–1.7375	0.4351
COVID-19 (hospitalized)	IVW	1.0031	0.9343–1.0770	0.9314
	MR Egger	1.0671	0.9081–1.2539	0.4744
	Weighted median	1.0176	0.9358–1.1065	0.6832
	Weighted mode	1.0258	0.9315–1.1297	0.6266
COVID-19 (very severe respiratory confirmed)	IVW	1.0012	0.9478–1.0576	0.9656
	MR Egger	1.0511	0.9194–1.2015	0.4985
	Weighted median	1.0082	0.9437–1.0771	0.8090
	Weighted mode	1.0230	0.9429–1.1100	0.6039
COVID-19 (critical illness)	IVW	1.0009	0.9557–1.0481	0.9703
	MR Egger	1.0219	0.9179–1.1377	0.7185
	Weighted median	1.0188	0.9619–1.0791	0.5254
	Weighted mode	1.0217	0.9534–1.0949	0.5758

IVW, inverse variance weighted; OR: odds ratio; CI: confidence interval.

**Figure 3:**
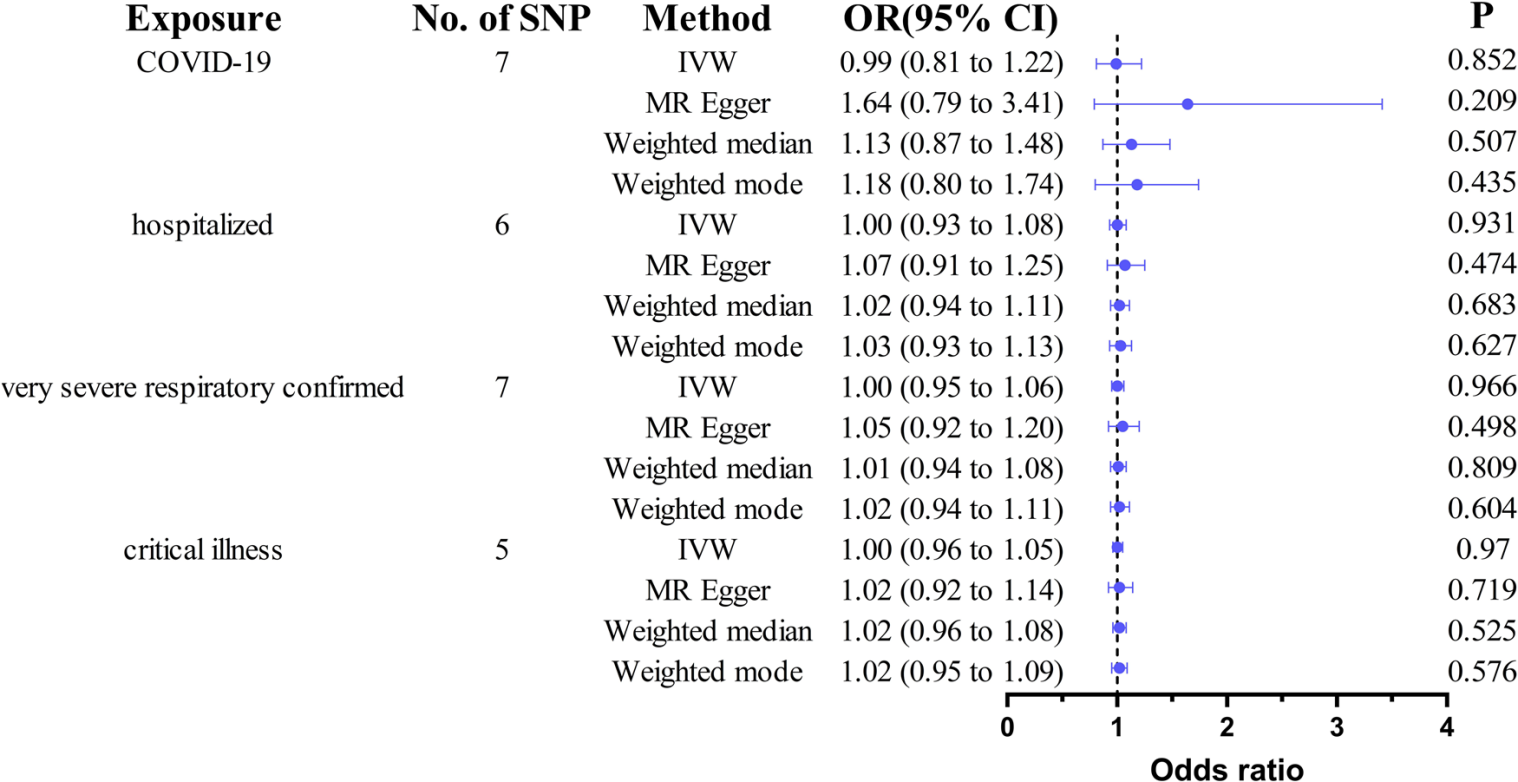
MR analysis of COVID-19 on spontaneous abortion. SNP, single-nucleotide polymorphisms; IVW, inverse variance weighted; MR, Mendelian randomization; OR: odds ratio; CI: confidence interval.

Sensitivity analyses using MR-Egger regression, weighted median, and weighted mode approaches yielded consistent results (all *p* > 0.05; Table 3 and Figure 3). No significant heterogeneity (Cochran’s *Q p* > 0.05) or directional pleiotropy (MR-Egger intercept *p* > 0.05) was detected (Table 4). The leave-one-out analysis confirmed that no single SNP had a disproportionate influence on the estimates (Figure S1).

**Table 4:** Sensitivity analyses for the MR of COVID-19 phenotypes on spontaneous abortion, including Cochran’s *Q* test for heterogeneity and the MR-Egger intercept test for pleiotropy

Exposure	*Q*/intercept	*p*-value
**COVID-19**		
Cochran *Q* test	*Q*	
MR Egger	2.3225	0.6767
IVW	4.2910	0.5083
Pleiotropy-test	Intercept	
	−0.04036	0.2333
**COVID-19 (hospitalized)**		
Cochran *Q* test	*Q*	
MR Egger	1.5374	0.8200
IVW	2.2370	0.8155
Pleiotropy-test	Intercept	
	−0.0162	0.4500
**COVID-19 (very severe respiratory -confirmed)**		
Cochran *Q* test	*Q*	
MR Egger	1.3158	0.9333
IVW	1.9245	0.9265
Pleiotropy-test	Intercept	
	−0.0156	0.4706
**COVID-19 (critical illness)**		
Cochran *Q* test	*Q*	
MR Egger	2.1641	0.5390
IVW	2.3413	0.6733
Pleiotropy-test	Intercept	
	−0.0089	0.7021

### 3.2 Impact of COVID-19 on male infertility

Genetic predisposition to COVID-19 infection was not found to be associated with the male infertility risk (IVW: OR = 0.7668, 95% CI = 0.3798–1.5484, *p* = 0.4590; Table 3). Similar null associations were also observed for COVID-19 severity phenotypes: for hospitalized COVID-19 patients: OR = 0.9085, 95% CI = 0.7100–1.1625, *p* = 0.4454; for cases of very severe respiratory-confirmed: OR = 0.9312, 95% CI = 0.7696–1.1267, *p* = 0.4633; and for patients with critical illness: OR = 0.94700, 95% CI = 0.8064–1.1121, *p* = 0.5063 (Table 5).

**Table 5:** MR estimates of the causal effect of COVID-19 phenotypes on the risk of male infertility

Outcome	Method	OR	95% CI	*p*-value
COVID-19	IVW	0.7668	0.3798–1.5484	0.4590
	MR Egger	0.0722	0.0078–0.6701	0.0687
	Weighted median	0.8117	0.3487–1.8893	0.6284
	Weighted mode	0.9141	0.3392–2.4635	0.8650
COVID-19 (hospitalized)	IVW	0.9085	0.7100–1.1625	0.4454
	MR Egger	0.9487	0.5423–1.6596	0.8624
	Weighted median	0.9163	0.6854–1.2249	0.5549
	Weighted mode	0.9311	0.6674–1.2991	0.6919
COVID-19 (very severe respiratory-confirmed)	IVW	0.9312	0.7696–1.1267	0.4633
	MR Egger	0.9860	0.6202–1.5675	0.9548
	Weighted median	0.9442	0.7488–1.1905	0.6273
	Weighted mode	0.9569	0.7200–1.2712	0.7716
COVID-19 (critical illness)	IVW	0.94700	0.8064–1.1121	0.5063
	MR Egger	0.9378	0.6457–1.3620	0.7580
	Weighted median	0.9560	0.7890–1.1585	0.6462
	Weighted mode	0.9702	0.7571–1.2432	0.8225

IVW, inverse variance weighted; MR, Mendelian randomization; OR: odds ratio; CI: confidence interval.

**Figure 4:**
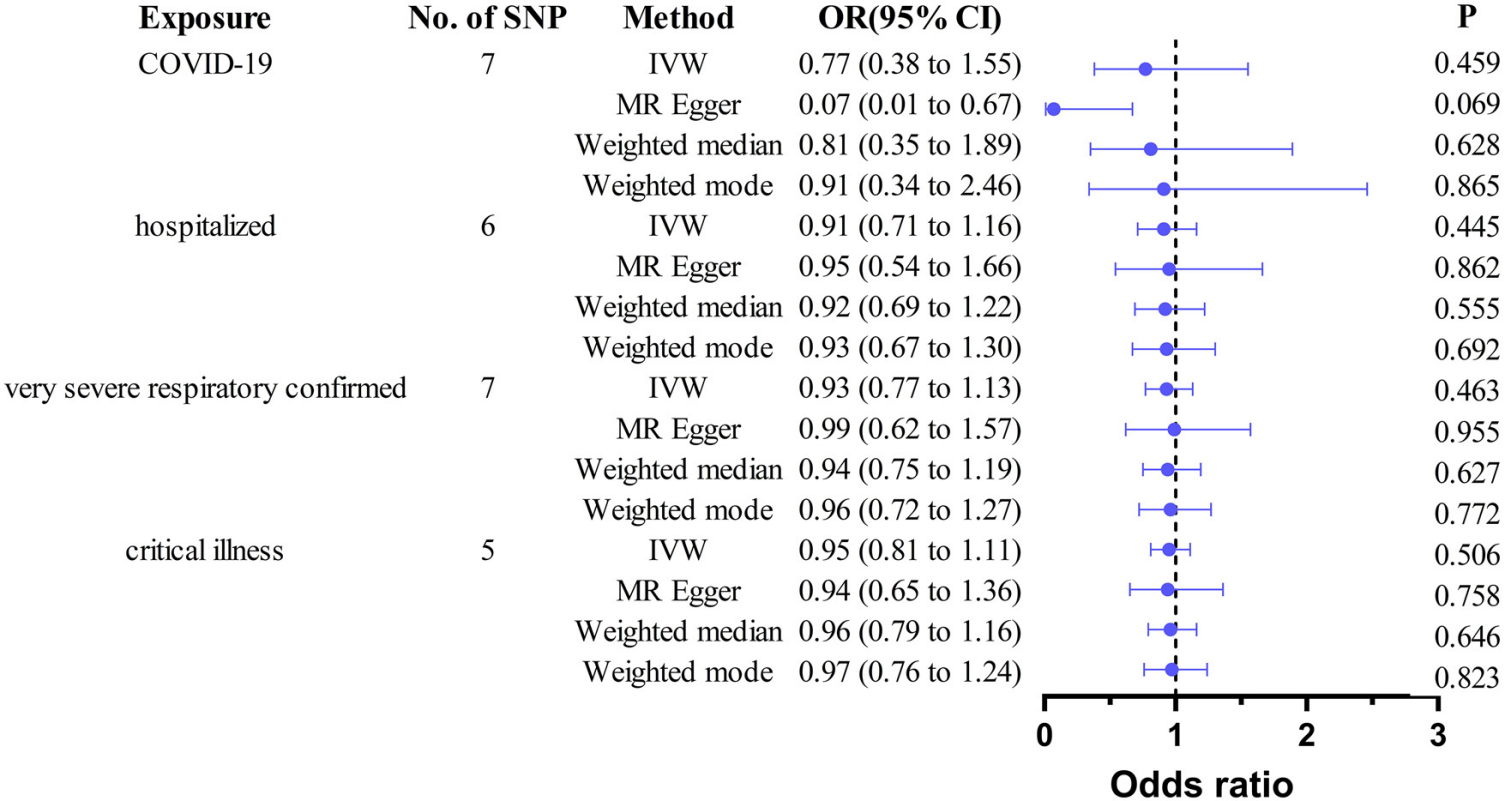
MR analysis of COVID-19 on male infertility. SNP, single-nucleotide polymorphisms; IVW, inverse variance weighted; MR, Mendelian randomization; OR: odds ratio; CI: confidence interval.

Sensitivity analyses and pleiotropy assessments (Cochran’s *Q p* > 0.05; MR-Egger intercept *p* > 0.05) further supported the robustness of these findings (Table 6 and Figure 4). Leave-one-out analysis revealed no influential SNPs (Figure S2).

**Table 6:** Sensitivity analyses for the MR of COVID-19 phenotypes on male infertility, including Cochran’s *Q* test for heterogeneity and the MR-Egger intercept test for pleiotropy

Exposure	*Q*/intercept	*p*-value
**COVID-19**		
Cochran *Q* test	*Q*	
MR Egger	3.5089	0.6220
IVW	8.1699	0.2259
Pleiotropy test	Intercept	
	0.2028	0.0833
**COVID-19 (hospitalized)**		
Cochran *Q* test	*Q*	
MR Egger	1.3465	0.8534
IVW	1.3751	0.9270
Pleiotropy test	Intercept	
	−0.0114	0.8740
**COVID-19 (very severe respiratory confirmed)**		
Cochran *Q* test	*Q*	
MR Egger	1.3795	0.9266
IVW	1.4498	0.9628
Pleiotropy test	Intercept	
	−0.0184	0.8013
**COVID-19 (critical illness)**		
Cochran *Q* test	*Q*	
MR Egger	0.9590	0.8112
IVW	0.9622	0.9155

## 4 Discussion

COVID-19 can induce dysfunction across various organ systems. While the respiratory system is predominantly affected, extrapulmonary manifestations involving organs like the testes and placenta have been documented [25]. However, the causal relationship between COVID-19 and reproductive complications (e.g., spontaneous abortion and male infertility) remains ambiguous due to the potential confounding factors and the inherent limitations of observational studies. Our two-sample MR analysis investigated this potential causality and found no significant genetic evidence linking COVID-19 susceptibility or severity to increased risks of male infertility or spontaneous abortion.

Currently, the majority of studies examining the association between COVID-19 and male infertility focus on the impact of COVID-19 on sperm quality. Li et al. detected the presence of SARS-CoV-2 in 6 out of 38 semen samples, with 4 patients in the acute phase and 2 in the convalescent phase [5]. Holtmann et al. found that sperm concentration and motility were significantly lower in recovered patients who had experienced fever compared to those who had not [26]. This implies that fever and disease severity may influence semen quality and potentially establish a link between COVID-19 and altered sperm parameters [26]. A prospective study indicated that COVID-19 infection was associated with male seminal inflammation and impaired seminal quality in the early stages of the disease, but these changes were partially restored after 1–2 sperm generation cycles [27]. Although relevant studies have established a correlation between COVID-19 infection and decreased sperm quality, the long-term implications for male infertility remain unclear. A recent clinical study by Sarier et al. directly compared the spermiogram parameters of infertile men before and during the first year of the COVID-19 pandemic [28]. They observed no significant differences in semen volume, the rates of normospermia, or the distribution of various pathological spermiogram findings, such as oligoasthenoteratozoospermia and asthenoteratozoospermia, between the pre-pandemic and pandemic periods [28]. This clinical observation and our MR results consistently show no causal link between genetic susceptibility to COVID-19 and male infertility risk. Despite the pandemic, there was no significant population-level decline in semen parameters or rise in male infertility. This is likely due to: (1) subclinical, short-term changes in semen parameters that do not lead to infertility and (2) sperm quality normalizing within one spermatogenic cycle (∼74 days) post-infection [29], rendering transient effects negligible for long-term fertility in genetically predisposed men. Notably, the GWAS sample size for male infertility was relatively small (680 cases), limiting statistical power and potentially missing small effects, though no significant associations, heterogeneity, or pleiotropy were found. In contrast, the spontaneous abortion analysis included a much larger number of cases (9,113), providing stronger evidence to support the null findings.

Evidence on the causal relationship between COVID-19 and spontaneous abortion remains limited. Although one retrospective study noted a higher miscarriage incidence (10.2%) among infected women, regression analysis showed COVID-19 was not a reliable predictor of pregnancy loss [13]. Most studies of viral impact in pregnancy focus on later trimesters, and early guidance recommended postponing assisted reproduction due to theoretical risks [30,31,32,33]. However, our MR analysis found no significant causal link between COVID-19 and spontaneous abortion. This is supported by an independent two-sample MR study using UK Biobank data, which also found no causal effect of hospitalized COVID-19 on miscarriage [34]. Further reinforcing these findings, a large case–control study found no association between COVID-19 vaccination during pregnancy and spontaneous abortion risk, regardless of dose timing or manufacturer [35]. Collectively, these complementary lines of evidence provide consistent reassurance, indicating that neither contracting COVID-19 nor receiving the recommended COVID-19 vaccine poses an increased risk of spontaneous abortion. The null findings align with several biological realities. First, SARS-CoV-2 primarily employs angiotensin-converting enzyme 2 (ACE2) and transmembrane serine protease 2 (TMPRSS2) as cellular entry receptors through its surface spike protein [36]. However, the ACE2 and TMPRSS2 expression in the human placenta, particularly during the first trimester, is minimal or restricted to specific non-critical cell types. Moreover, their expression is notably absent or negligible in syncytiotrophoblasts, which mediate maternal-fetal exchange and barrier functions [37]. This spatially constrained and developmentally limited receptor expression drastically reduces the likelihood of placental SARS-CoV-2 infection and subsequent vertical transmission during early gestation. Second, first-trimester pregnancy loss is predominantly driven by embryonic chromosomal abnormalities (aneuploidy) [38,39]. While maternal infection can be a contributing factor in some cases, our genetic findings do not support SARS-CoV-2 as a major causative agent. Furthermore, an important avenue for future research will be to investigate the potential joint impact of dual parental COVID-19 infection (both maternal and paternal) around the time of conception on early pregnancy outcomes, which was beyond the scope of this study. This comprehensive understanding is crucial for alleviating public anxiety, particularly among pregnant women who are concerned about early pregnancy loss. It also serves as a solid foundation for supporting evidence-based public health recommendations regarding both infection prevention and management during the ongoing pandemic.

This study has several limitations that warrant consideration. First, residual bias from weak instruments or undetected horizontal pleiotropy, inherent to MR, cannot be fully ruled out despite robust sensitivity analyses. Second, our findings are based on European-ancestry GWAS data, limiting their generalizability to other populations. Third, our analysis did not differentiate between SARS-CoV-2 variants, which may have differing pathogenic effects. Fourth, the statistical power for the male infertility analysis was limited by a relatively small case number (*n* = 680), potentially obscuring subtle causal effects. Future studies incorporating diverse populations, larger male infertility cohorts, and variant-specific data are warranted to further elucidate the long-term reproductive implications of SARS-CoV-2 infection.

In conclusion, our MR study provides robust genetic evidence against a causal relationship between COVID-19 and an increased risk of male infertility or spontaneous abortion. These findings have key clinical implications. Clinicians can reassure convalescent men that any infection-related semen alterations are likely transient and reassure pregnant women that a first-trimester COVID-19 infection is not a direct indication for termination. Clinical management should be guided by standard obstetric and urological indications not solely by COVID-19 infection status.

## Supplementary Material

Supplementary Figure
