# Supplementary material for: Can coronavirus disease 2019 affect male fertility or cause spontaneous abortion? A two-sample Mendelian randomization analysis
Source: Open Life Sci. 2025 Oct 30;20(1):20251188. doi: 10.1515/biol-2025-1188 (PMC12596030; doi:10.1515/biol-2025-1188)
Supplement: Supplementary Figure [file biol-2025-1188-sm.pdf]

Supplementary material

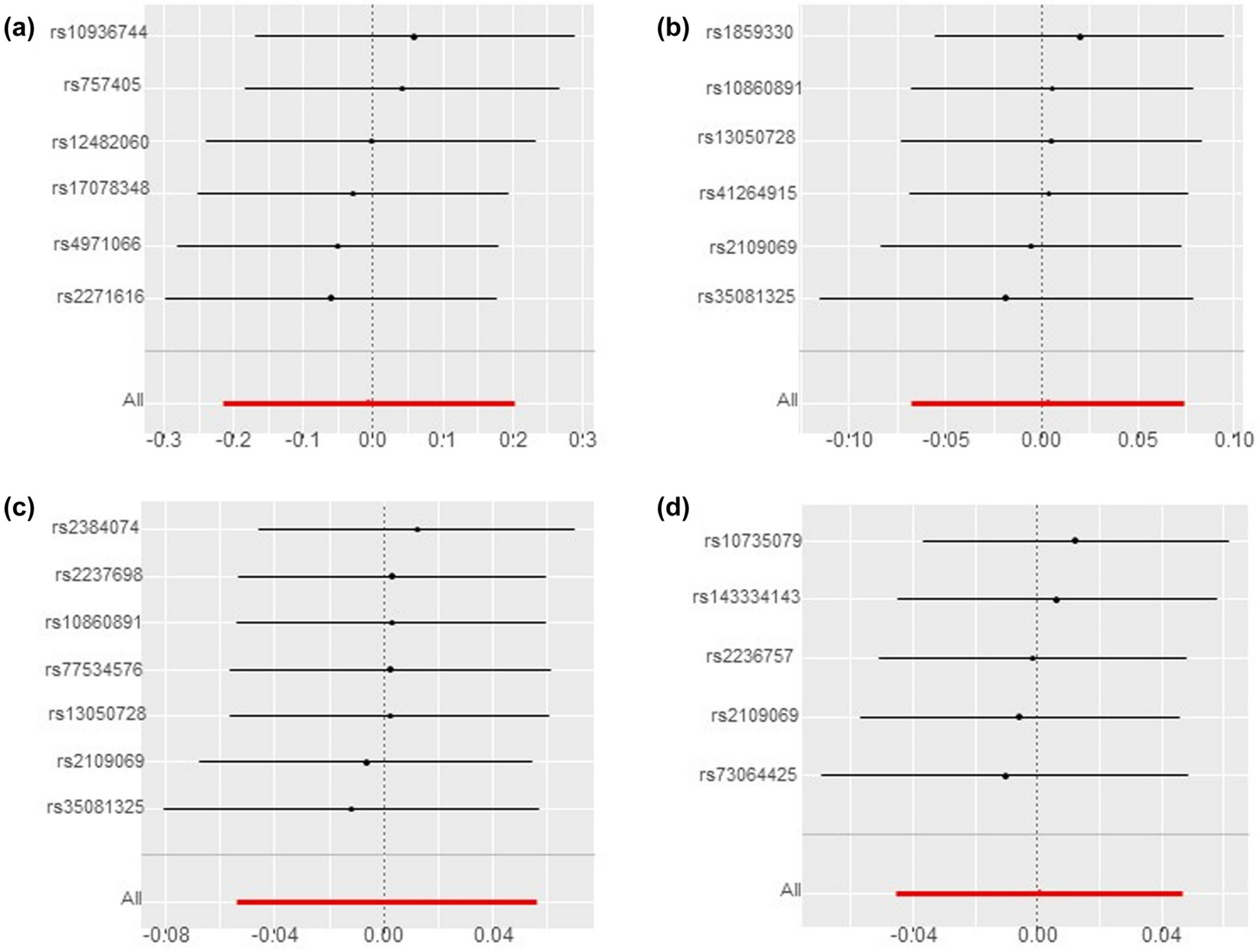

Figure S1: Leave-one-out sensitivity analysis for COVID-19 on spontaneous abortion. (a) Leave-one-out sensitivity analysis on COVID-19 and spontaneous abortion; (b) Leave-one-out sensitivity analysis on COVID-19 (hospitalized) and spontaneous abortion; (c) Leave-one-out sensitivity analysis on COVID-19 (very severe respiratory-confirmed) and spontaneous abortion; (d) Leave-one-out sensitivity analysis on COVID-19 (critical illness) and spontaneous abortion;.

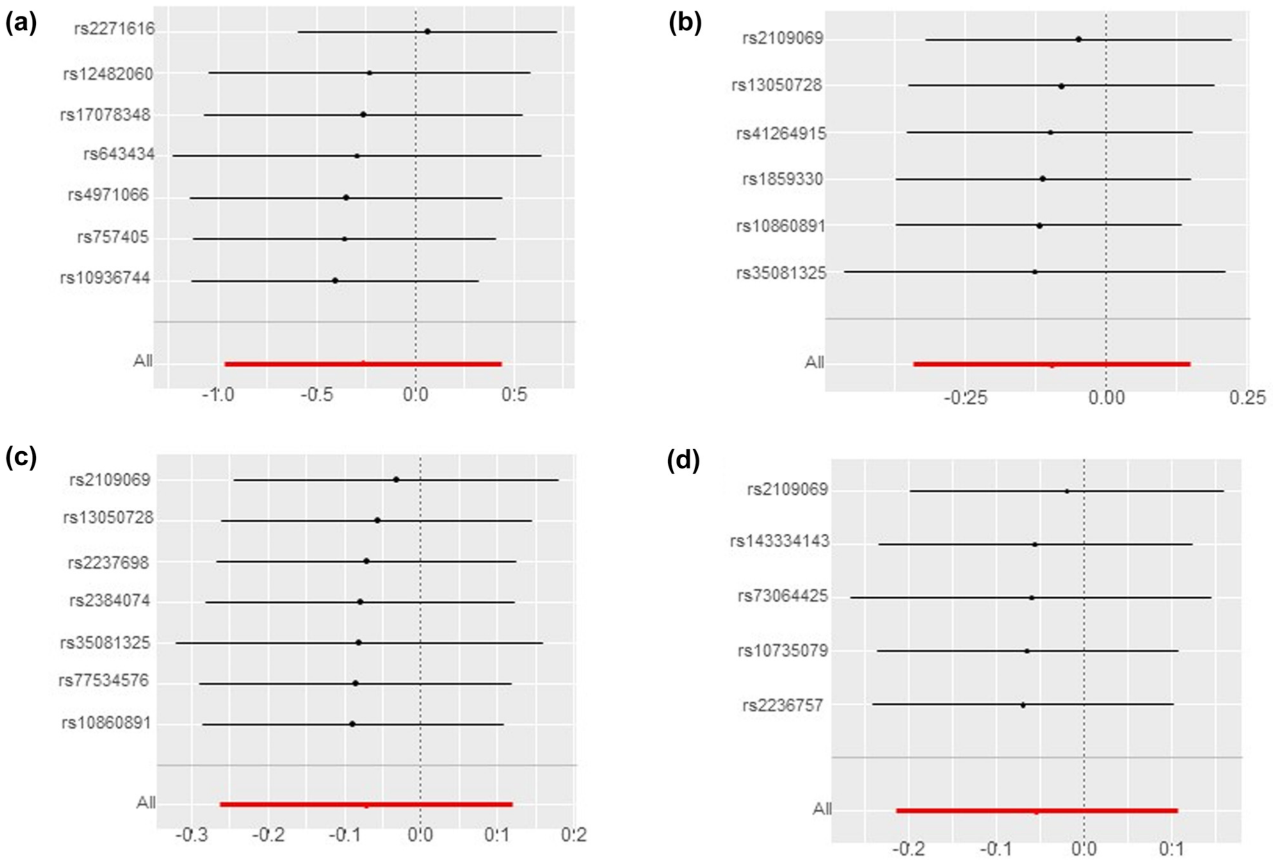

Figure S2: Leave-one-out sensitivity analysis for COVID-19 on male infertility. (a) Leave-one-out sensitivity analysis on COVID-19 and male infertility; (b) Leave-one-out sensitivity analysis on COVID-19 (hospitalized) and male infertility; (c) Leave-one-out sensitivity analysis on COVID-19 (very severe respiratory-confirmed) and male infertility; (d) Leave-one-out sensitivity analysis on COVID-19 (critical illness) and male infertility.
